# Optically and electrically driven nanoantennas

**DOI:** 10.3762/bjnano.11.136

**Published:** 2020-10-07

**Authors:** Monika Fleischer, Dai Zhang, Alfred J Meixner

**Affiliations:** 1 Institute for Applied Physics and Center LISA⁺, University of Tübingen, Auf der Morgenstelle 10, 72076 Tübingen, Germany; 2 Institute of Physical and Theoretical Chemistry and Center LISA⁺, University of Tübingen, Auf der Morgenstelle 18, 72076 Tübingen, Germany

**Keywords:** active plasmonics, electrically driven nanoantenna, gap antenna, nanoantenna, nanofabrication, nanospectroscopy, nano-photonics, optical antenna, second harmonic generation, sensing, scanning tip, surface-enhanced infrared absorption (SEIRA), surface-enhanced Raman spectroscopy (SERS), tip-enhanced Raman spectroscopy (TERS), tunnel junction

## Editorial

Optical antennas ^+^ serve to confine the energy of photons transported by a light wave to a tiny volume much smaller than the wavelength; or reversely, to convert the energy of an evanescent field that oscillates at optical frequencies to a traveling electromagnetic wave that can be observed in the far field. Optical antennas are key elements in nano-optics, bridging the gap between the dimension of an optical wavelength (several hundreds of nanometers) and the size of elementary quantum emitters such as single atoms, molecules, etc. (a few nanometers to below 1 nm). Nanoantennas have been under examination for the past few decades in view of their attractive fundamental properties, while the rapid development of nanofabrication techniques has opened up possibilities to create more and more sophisticated shapes and configurations with increasing control over their optical performance [1–4]. The strong local near-field enhancement by plasmonic nanoantennas is being harnessed for high sensitivity, high-resolution optical nanospectroscopy techniques [5], such as surface-enhanced or tip-enhanced Raman spectroscopy (SERS or TERS) [6–15], as well as for (bio-)sensing applications [16–18]. The integration of nanoantennas can lead to enhanced functionality for optoelectronic devices, nano-light sources, light amplification, or hybrid systems in combination with nanoemitters or two-dimensional materials [19]. Under excitation with state-of-the-art lasers, ultrafast effects can be observed as well as a plethora of nonlinear characteristics [20]. Recently, also electrically driven nanoantennas have been demonstrated [21–25], which are an important milestone towards on-chip integration, device-to-device communication, and bilateral transduction between electrons and photons [26].

An optical gap antenna typically consists of two nanostructures with a nanometer gap in between. Optical excitation induces a coupled plasmon oscillation along the two antenna parts, which can lead to large surface charge oscillations on the opposite surfaces confining the gap, and a field in the gap that is enhanced by several orders of magnitude with respect to the incident field [27]. The gap field is particularly strong when the particle plasmons are in resonance. The enhanced local field and the enhanced optical mode density can, for example, enhance the excitation and/or the emission rate of a quantum system that is positioned in this near-field region [28].

Both the enhanced local field and optical mode density are of great practical use for local spectroscopy with extremely high spatial resolution. They enable boosting the intrinsically low optical signal of a very small number of molecules by orders of magnitude with respect to any surrounding molecules, which can be the source of an overwhelming background signal. This principle applies to virtually any kind of optical spectroscopy. Prominent examples are SERS and TERS, where the intrinsically small Raman scattering cross-section is enhanced by several orders of magnitude, making single-molecule spectroscopy feasible. These spectroscopic techniques have shown tremendous progress in the last two decades [29–32].

Under high-power excitation of nanoantennas, higher order contributions to the particle polarization are no longer negligible, and nonlinear effects become apparent. Due to their inherent symmetry breaking, nanoantennas can act as local sources of second harmonic generation (SHG) with attractive applications in low-background medical imaging or nanolasers [33].

Electrically driven optical antennas emit light when a bias voltage is applied to the contacted antenna arms that are forming a tunnel junction. Inelastic electron tunneling through the gap excites gap–plasmon oscillations leading to the emission of photons as a consequence of radiative plasmon relaxation [34–36]. The relaxation depends on how the gap modes couple and can hence be controlled by the design of the antenna. The combination of an electrically driven antenna with optical excitation is a very promising but not yet well explored subject. Energy-level engineering in the gap by introducing molecules into the tunnel junction provides an additional handle to modulate photon emission from an electrically controlled optical antenna. Light emission by tunneling through a single molecule opens the door to combine electronics and quantum optics for a new class of quantum devices [37].

The concept, realization and prospective applications of such optically and electrically driven antennas are explored in the *Beilstein Journal of Nanotechnology* Thematic Issue, “Optically and electrically driven nanoantennas”.

In most applications for ultrahigh sensitivity sensing the central feature for tightly confining and enhancing the optical near-field is a narrow gap between two metallic nanoparticles or tips. Creating such gaps to obtain a controlled distribution of hotspots, for example, on a chip, is no trivial task. This has been pursued using top-down approaches such as thin film deposition and nanopatterning, as well as bottom-up approaches such as chemical synthesis and self-assembly [38]. Gap sizes may range from a few tens of nanometers down to sub-nanometer tunnel junctions, where the classical description of the plasmonic behavior breaks down [39–40]. Here, several approaches to create low-cost, large-area SERS substrates that exhibit homogeneous Raman intensity enhancement are introduced. The substrates are based on quasi-hexagonally ordered gold particles prepared by block-copolymer micellar nanolithography and electroless deposition [41], or on dense silver island films created by pulsed laser deposition [42] or physical vapor deposition [43]. In [44], individual plasmonic nanotags are prepared by coating gold nanoparticle clusters with Raman reporters. This work explores the minimum number of tags required for obtaining a SERS signal under operation conditions. Moving further towards the (near-) infrared regime, different antennas are employed in a surface-enhanced infrared absorption (SEIRA) configuration [45]. Here the aim is to detect low concentrations of semiconductor nanocrystals through maximum local enhancement of their optical phonon response. High local field enhancement is likewise required in TERS experiments, where a tip with a hotspot located at its apex is scanned across a sample surface. The performance of a TERS measurement is closely related to the quality of the tip [46]. Therefore, researchers are on the lookout for nanotips that can be prepared fast and with reproducible properties, and at the same time, aim for ever higher field enhancement and localization to improve the sensitivity and spatial resolution of the TERS information. In [47], an earlier protocol for etching thin gold wires to a sharp tip is refined to a two-step procedure with high throughput. A self-assembly approach to create a dumbbell antenna consisting of a 40 nm and an 80 nm Au nanoparticle at a scanning tip is shown in [48] and applied for single molecule sensitivity imaging. The advantage of applying TERS for revealing local structural properties is illustrated in [49], where crystalline and amorphous regions within core–shell silicon nanowires are discerned with an optical resolution of a few nanometers. This study further demonstrates that it is possible to combine polarization angle-resolved experiments with a TERS setup, which has been rarely pursued so far.

In the hot topic area of active plasmonics, reversible changes in the refractive index of the environment of a plasmonic system, for example, by liquid crystals or thermosensitive polymers, allow for actively switching the plasmonic properties [50–51]. A recent example of the strong coupling between silver nanorods and photochromic molecules is demonstrated in [52].

Due to the high local field intensity in the gap of a nanodimer, strong polarization of the particle, and consequently, strong SHG from the particle surface, may be expected. However, owing to the polarization and far-field interference, a silencing of the SHG is observed for decreasing gap sizes [53]. This effect is further studied in [54] using the surface integral equation method. On the other hand, the generation of higher harmonics by a dielectric nanostructure can be boosted by forming a hybrid antenna. In the case of [55] an AlGaAs nanopillar, which has an anapole mode resonant with the pump wavelength, is encompassed by a gold ring. The field enhancement by the plasmonic structure is shown to lead to almost two orders of magnitude improvement of the SHG and a sizeable third-harmonic generation from the pillar. The fact that nonlinear effects can already be observed in the absorption and scattering of single gold nanoparticles at much lower laser intensities is demonstrated in [56]. Using the so-called *x*-scan technique, a nanoparticle is scanned through a confocal laser spot, and power-dependent modifications are observed in the resulting point-spread function profile.

Last but not least, the electrically driven generation of photons is explored in [26] and [57]. In [26] the emission of light from electromigrated in-plane tunnel junctions is observed, where the feed-gap is shown to couple to propagating modes in waveguides with up to 30% efficiency. Making use of propagating surface plasmon polaritons (SPPs), directional light beams are created in [57]. The SPPs are excited by inelastic tunneling from a scanning probe. The probe is positioned in the focus of an elliptical structure, which is formed by a slit in a gold film. By defining the dimensions of the ellipse, it becomes possible to control the directionality of the emitted light beam.

The contributions collected in this Thematic Issue thus highlight current and emerging directions in the fast-moving field of optically and electrically driven nanoantennas. In the current age of photonics, such elements may prove essential in further expanding the functionality of electrical devices by integration with photonic functionalities. We sincerely thank all colleagues who contributed to this Thematic Issue with their time and research results.

Monika Fleischer, Dai Zhang and Alfred J. Meixner

Tübingen, September 2020
